# Investigating Plant Micro-Remains Embedded in Dental Calculus of the Phoenician Inhabitants of Motya (Sicily, Italy)

**DOI:** 10.3390/plants9101395

**Published:** 2020-10-20

**Authors:** Alessia D’Agostino, Antonella Canini, Gabriele Di Marco, Lorenzo Nigro, Federica Spagnoli, Angelo Gismondi

**Affiliations:** 1 Department of Biology, University of Rome “Tor Vergata”, Via della Ricerca Scientifica 1, 00133 Rome, Italy; d.agostino@scienze.uniroma2.it (A.D.); gabriele.di.marco@uniroma2.it (G.D.M.); 2 Department Italian Institute of Oriental Studies—ISO, Sapienza University, Piazzale Aldo Moro 5, 00185 Rome, Italy; lorenzo.nigro@uniroma1.it (L.N.); federica.spagnoli@uniroma1.it (F.S.)

**Keywords:** tartar, secondary metabolites, gymnosperm products, palaeodiet, nutritional ecology, Punic archaeology

## Abstract

Plant records reveal remarkable evidence about past environments and human cultures. Exploiting dental calculus analysis and using a combined approach of microscopy and gas chromatography mass spectrometry, our research outlines dietary ecology and phytomedicinal practices of the ancient community of Motya (Sicily, eight to sixth century BC), one of the most important Phoenician settlements in the Mediterranean basin. Micro-remains suggest use or consumption of Triticeae cereals, and animal-derived sources (e.g., milk and aquatic birds). Markers of grape (or wine), herbs, and rhizomes, endemic of Mediterranean latitudes and the East, provide insight into the subsistence of this colony, in terms of foodstuffs and phytotherapeutic products. The application of resins and wood of Gymnosperms for social and cultural purposes is hypothesized through the identification of Pinaceae secondary metabolites and pollen grains. The information hidden in dental calculus discloses the strong human-plant interaction in Motya’s Phoenician community, in terms of cultural traditions and land use.

## 1. Introduction

Over the last decade, the analysis of microparticles entrapped in dental calculus has contributed significantly to the knowledge about ancient subsistence systems and human interaction with different environments [1,2,3,4,5]. Indeed, animal and plant micro-remains, deliberately or accidentally ingested or inhaled during daily activities and calcified in mineralized plaque, provide information about nutrition and other aspects of lifestyle [6,7,8,9,10]. The body of evidence regarding ancient use of plant species is continually increasing. It demonstrates the key role that plants have represented for past communities, in food practices and cultural traditions [11,12,13,14,15,16]. However, the contribution of plants to the human diet remains difficult to estimate by using dental calculus, due to archaeological matrix’s multifactorial aetiology. Indeed, several factors, such as genetic predisposition, oral hygiene practices, levels of inorganic salts contained in saliva, and oral pH, contribute to calculus growth [17,18]. Furthermore, some types of micro-debris (e.g., starches) are subjected to diagenetic changes, gelatinization, and mechanical destruction, which can make identification even more difficult or impossible. For these reasons, not all microparticles (e.g., starches and pollen grains) have the same likelihood of conservation inside the ancient dental plaque [19]. Taxonomic identification of plant remains that are entrapped in this powerful archaeobotanical matrix is often difficult and the low amount of calculus available per individual limits the analyses that can be performed. Despite these shortcomings, dental calculus still remains one of the most informative fossilized records available with regards to ancient lifestyles [1,6,20,21,22].

Concerning Phoenician archaeobotanical scientific research, Southern Italy and Sicily are still underinvestigated [23,24,25]. In this respect, we aim to contribute by reconstructing the use of plant resources by the Phoenician community of Motya (Sicily, Italy, eight to sixth century BC), one of the most important Phoenician sites in the Western Mediterranean. Dental calculus analysis allowed us to reconstruct dietary ecology and phytotherapeutic knowledge of this population, for whom archaeobotanical findings have remained yet undocumented.

### Archaeological and Historical Context

**Figure 1:**
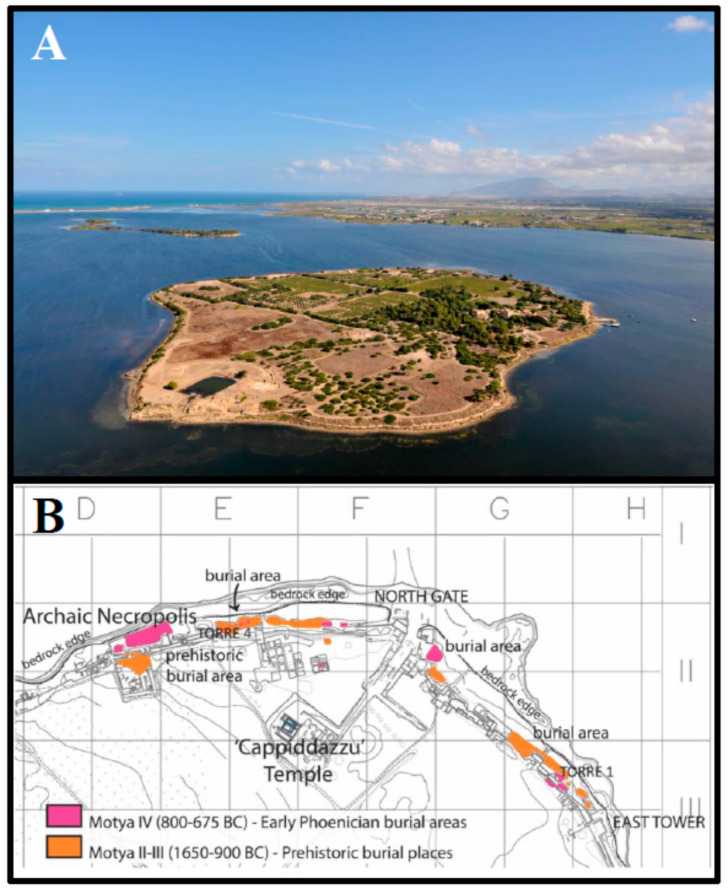
Archaeological site of Motya. (**A**) South view of San Pantaleo island located at the center of the Marsala Lagoon (Trapani, western tip of Sicily, Italy) (archive of Sapienza Archaeological Expedition at Motya); (**B**) Northwestern sector of Motya [34], the Phoenician burial areas were highlighted in pink.

The island of Motya, nowadays known as San Pantaleo (37°52′06″ N 12°28′07″ E), is located at the western tip of Sicily (Italy) facing the Marsala Lagoon. It was a prominent prehistoric site and one of the major Phoenician settlements in the Mediterranean area (Figure 1A). Its archaeology has shed light on the history of the Middle Sea during the second and the first millennium BC. Moreover, it has also provided useful insight into local ancient communities, offering a vivid picture of interactions and cultural exchanges that occurred in this geographic area. In fact, Sicily holds a central position in the Mediterranean Sea, because it is a hub between different civilizations. From the second half of eighth century BC, the Phoenician colony of Motya started controlling the trade between the Western and Eastern Mediterranean, together with Carthage [26]. The Phoenicians have always been considered to be skilled merchants who extended their commercial networks throughout the Mediterranean Sea. The livelihood of their cities and seaports was based on an exchange economy [27,28]. Fish, salt, and clays were suggested as the most exploited raw materials by Motya’s Phoenician settlers [26].

From prehistorical times, the burial place of Motya’s inhabitants was the island’s northeastern strip, whose natural limestone-marl bedrock contained caves that were convenient for burials [29,30,31]. These caves became the setting of Middle Bronze Age tombs. Subsequently, with the arrival of the early Phoenicians, they were reused to house urns and funerary assemblages. In the 1970 and 80s, Antonia Ciasca unearthed human remains, among the filling layers of the city walls. These were built around the city in the mid sixth century BC, destroying the tombs of the earlier necropolis, which had extended from the northwest coast of the island to the eastern side (Figure 1B) [32,33]. The necropolis provides evidence of several types of burial rites, including the inhumation in wooden or stone sarcophagi. In Motya, evidence of the latter practices dates only from seventh century BC; previously, incineration was the prevalent custom. The material examined in our own study belongs to the Phoenician inhabitants of Motya (sixth century BC) [34].

## 2. Results

### 2.1. Optical Microscopy (OM) Analysis

We used optical microscopy to observe 69 starch granules, five pollen grains, one fragment of feather barbule and two plant trichomes in the dental calculus samples (Table 1).

Descriptions of micro-remains and animal micro-debris detected in dental calculus by optical microscopy are as follows:

#### 2.1.1. Starch Granules

**Figure 2:**
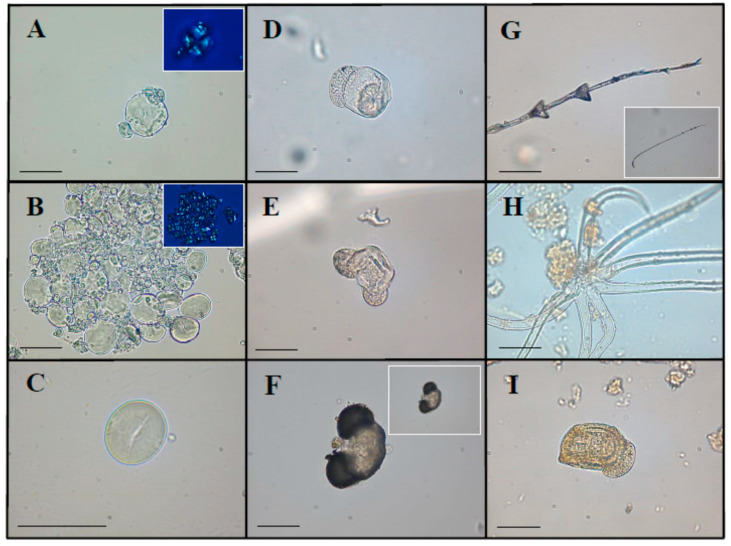
Mosaic of representative micro-debris found in Phoenician dental calculus by optical microscopy. (**A**) Triticeae starch granules and relative polarized image; (**B**) Aggregate of Triticeae starch grains and relative polarized images; (**C**) Modern reference of *Triticum dicoccum* L. starch; (**D**,**E**) Gymnosperm pollen grains; (**F**) Modern reference of gymnosperm pollen; (**G**) Fragment of feather barbule typical of Anseriformes; (**H**) Multiradiate non-glandular trichomes and unidentified fibers; (**I**) Damaged gymnosperm pollen grain. Each scale bar represents 40 µm.

Only one starch morphotype was identified and described, according to standard morphological and metric parameters reported in literature [35,36]. These characteristics included length and width, shape, presence or absence of lamellae, hilum position (e.g., centric or eccentric), fissures, and birefringence (namely appearance and projection of the characteristic “Maltese cross” under cross-polarized microscope). These grains possessed a bimodal distribution, typical of most grasses belonging to Triticeae tribe (e.g., *Triticum* spp. L., *Secale* spp. L., and *Hordeum* spp. L.). In particular, the diagnostic starches were oval to sub-rounded in two-dimensional (2D) shape (15–43 µm in length and 10–35 µm in width) with central and distinct hilum (associated with fissure) and concentric, complete and distinct lamellae (Figure 2A,B).

#### 2.1.2. Pollen 

Five pollen granules were recovered in four dental calculus samples and attributed to gymnosperms (Figure 2D,E,I) [37,38,39]. These ancient micro-remains displayed morphological traits consistent with Pinaceae pollen, most likely *Pinus* sp. L. Indeed, they appeared to be bisaccate and oblate monads with an elliptic corpus in polar and equatorial view. Sacci were spherical and narrowly connected to the corpus (attachment area 25–37 µm). Pollen dimensions were 67–75 µm in diameter and 47–51 µm in height.

#### 2.1.3. Other Plant Micro-Remains

Two trichomes were retrieved from the calculus matrix (Figure 2H). These multicellular and compound trichomes were characterized by 7–8 subulate branches, with some differences in length of the individual arms (164–276 µm). The scarcity of details prevented us from establishing this morphology as diagnostic. In fact, several plant species present stellate trichomes on the adaxial surface of their leaf epidermis, in line with the literature [40,41,42,43]. Therefore, it was not possible to identify these micro-remains at the taxonomic level.

#### 2.1.4. Animal Micro-Debris

Calculus flakes of the individual buried in Tomb 8 contained one fragment of a feather barbule (Figure 2G). This microparticle (857.2 µm in length) presented diagnostic distal triangular-shaped nodes (located toward the distal one-third of the barbule) and prongs typical of waterfowl barbules; as a result, it was attributed to the Anatidae family (Anseriformes order) [44].

### 2.2. Gas Chromatography and Mass Spectrometry (GC-MS) Analysis

The chromatographic approach was applied on ancient dental calculus to detect molecular markers of substances ingested and inhaled by the individuals. The list of these chemical compounds is reported in Supplementary Material 1. Although ever-present in GC-MS profiles, *n*-alkanes and *n*-alkenes (C_6_ to C_33_) are not shown in the list because they were widespread and not taxonomically specific. In particular, the possibility of hypothesizing their original source is problematic, due to the dental calculus’s multifactorial aetiology. They most probably derived from degradation of oral bacteria and dietary components, such as unsaturated and saturated fat- and oil-derived acyl lipids and higher plant waxes [12,45,46,47].

The unusual detection of fatty acid profiles in some calculi led us to reflect upon their potential origin, despite their being ubiquitous components of organic matter [48,49,50]. Odd chain fatty acids, usually occurring at low quantity in plant oils and animal fats, had previously been used as indicators of bacterial or ruminant lipids [47,51,52]. The presence of short chain fatty acids could represent degradation forms of medium and long chain acids, despite traces of them being found in many plant and animal lipids [51]. Finally, long chain fatty acids were presumed to have derived from leaves, fruits, and fatty plant and animal tissues [49,50]. In our study, the ratio between palmitic and stearic acids, both retrieved in almost all the samples, was not evaluated since, in calculus, residues could have been derived from multiple sources. Another reason is that fatty acids, as well-known, oxidize at different rates [47]. These biomolecules, in the form of methyl esters, were attributed to the heating process of lipids (i.e., fats/oils) and were considered to be bacterial markers [12,53,54]. Long-chained polyunsaturated fatty acids (PUFAs) (e.g., docosahexaenoic acid (DHA) and eicosapentaenoic acid (EPA)) and their derivatives were detected in some samples. Dried fruits (e.g., nuts) contain PUFAs, but oily fish, algae, and molluscs are the richest dietary sources of these molecules [55,56]. For its part, the identification of lactose, occurring in milk and its derivatives, probably testified to the intake of dairy products [57].

Several samples showed terpenic compounds, such as gurjunene, citronellol, levomenthol, limonenol, ocimene, anisole, and salvialenone, which are characteristic of plants (e.g., species belonging to Apiaceae, Lamiaceae, and Asteraceae) [58,59,60,61]. Many molecular markers typical of gymnosperm resins (i.e., sabinol, junenol, alpha-pinene, cedrol, abietic acid, and dehydroabietic acid) were found in three specimens. In particular, abietane compounds are the main components of Pinaceae resins [62,63,64,65].

Chromatographic analysis also revealed the presence of tartaric acid, abundant in *Vitis vinifera* L. products, and the following three sesquiterpene derivatives typical of plant rhizomes: (i) spirojatamol, synthesized in *Nardostachys* sp. DC. [66,67]; (ii) curdione, one of the major components of *Curcuma* sp. L. [68,69]); and (iii) alpha-acoradiene, contained in underground storage organs of *Curcuma* sp. L. and *Acorus* sp. L. [70,71].

## 3. Discussion

In the present research, we aimed at obtaining archaeobotanical evidence from the dental calculus of a group of individuals buried in Motya, one of the Western Mediterranean’s most important Phoenician colonies, located in Sicily (Italy) and dated to the eight to sixth century BC.

Understanding how different foods contributed to the diet of Motya’s Phoenician community is still problematic. Indeed, no direct correlation exists between the presence of animal and plant micro-remains in dental calculus, the amount of foods entering via diet (or for medicinal and cultural purposes), and the moment or place, in which those were consumed by the individuals [18]. For this study, archaeobotanical investigations on plant macro-remains found in this archaeological site have not yet been completed. However, the present preliminary data from dental calculus could shed light on the main plant species used by the community, or species with which individuals came into contact involuntarily. As stated above, the Phoenicians had a reputation as skilled merchants, thus, some micro-remains may not come from activities carried out in Motya. However, due to the multifactorial aetiology of the tartar and the lack of information about each individual’s identity, it is impossible to assess where and when these plant remains were trapped in the calculus. The morphological approach performed in this study revealed only starches of the Triticeae tribe (e.g., wheat, barley, and rye), suggesting the consumption of these cereals as a source of carbohydrates. Grain was the main component of the daily nutrition both for Near East populations, throughout the Bronze and Iron Ages, and for the inhabitants of the Mediterranean’ coastal regions. Indeed, the Phoenician diet, both in the mother country and in the colonies, was based on these crops, as confirmed by carpological remains from archaeological sites of Tell el-Burak in Lebanon, Duos Nuraghes in Sardinia, and Utica in North Africa [28,72,73]. Barley and wheat, mixed with pulses, were usually consumed as a porridge, or in the form of bread and flat cakes [74]. In Motya’s case, Phoenicians established contact with the autochthonous populations inhabiting the rich and fertile Sicilian hinterland, such as the Elyms distributed along the Belice Valley and in the Trapani area. This relationship consisted not only of military alliances, as widely documented by classical sources [75,76], but also in commercial exchanges, aimed at supplying the island with cereals, such as barley (*Hordeum* sp.) and wheat (*Triticum* sp.), as well as wild game [77].

The processing of starchy sources and the several mastication patterns may increase the exposure of starch to cooking liquids and alpha amylase activity, influencing granule survival and structured arrangement of amylose and amylopectin polymers [5,18]. How dental calculus builds up is still understudied and knowledge about the different pathways of microparticle incorporation remains problematic [7,78].

Although plant hairs are some of the most common debris in air-borne particulate matter (as pollen), only rarely were these plant micro-remains found in dental calculus [9]. Trichome identification is not a common area in dental calculus research; thus, our findings are unique and emphasize ancient tartar’s potential for also capturing non-nutritional evidence. Nevertheless, it was impossible to identify this type of micro-remains at the species level. Since two trichomes with the same morphology were found in two individuals from the studied community, the possibility that their plant source had been voluntary handled and used for ethnobotanical purposes cannot be excluded. However, the hypothesis of accidental inhalation must also be taken into consideration.

The fragment of barbule potentially derived from non-dietary activities, such as dust generated while plucking or chewing epidermal fragments of birds. In this regard, it is worth remembering that the Marsala Lagoon (northwestern coast of Sicily) is one of most important sites for Mediterranean bird migratory routes. This area hosts both resident and migratory species that take advantage of this favorable environment for nesting and wintering [79]. Game and small birds were commonly consumed by Motya’s inhabitants, as recorded by findings of sacred meals in the Temple of the Kothon [80,81]. However, the main hypothesis is that the fragment of barbule was accidentally inhaled by the individual.

**Figure 3:**
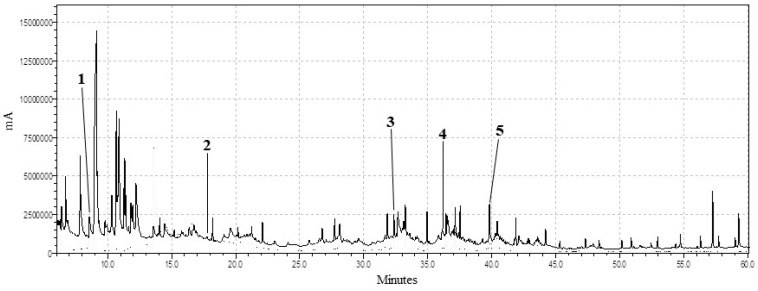
Total ion chromatogram. Peaks corresponding to chemical markers identified in the dental calculus of one individual (Burial 20) were indicated (1, alpha-pinene; 2, sabinol; 3, hexadecanoic acid; 4, octadecanoic acid; 5, dehydroabietic acid).

The analytical techniques applied on the ancient dental plaque both provided evidence of coniferous exudates, or rather water-insoluble mixtures of terpene compounds derived from gymnosperms. In some samples, such as at Burial 12, 20 and 38, calculus flakes preserved both pollen grains and molecular markers typical of Pinaceae (e.g., dehydroabietic acid) (Figure 2 and Figure 3) [82,83]. The identification of ancient pollen can be challenging, especially if its conservation is inadequate. It is important to remember that the presence of pollen in the dental calculus can also derive from accidental inhalation linked to breathing [7]. The palynomorphs characterized in this study, given their good state of preservation, were tentatively ascribed to the *Pinus* L. species. With respect to these findings, written sources and archaeological data evidence the Phoenician merchants’ interest in exporting and using coniferous wood for temple decoration and shipbuilding, and Pinaceae resins for pottery waterproofing, wine flavoring, and avoiding the conversion of wine into vinegar [84,85,86,87,88]. However, the application of gymnosperms by this civilization was not limited to our bioarchaeological micro-remains. Oil bottles and incense burners found in Motya’s temples and sacred areas indicated the use of plant essential oils and perfumed resins for medicinal (e.g., personal hygiene) and religious (e.g., gift to the deity) purposes [89,90]. Additionally, in the necropolis of Tyre Al-Bass, charcoal from *Pinus pinea* L. was found inside the graves, which had been part of a funerary ritual. Indeed, this type of wood was burnt before sealing the tomb, to ritually purify the deceased with its aromatic smoke [91].

Phoenicians were capable oenologists who introduced viticulture and wine-making skills in their Mediterranean colonies, such as Carthage and pre-imperial Rome sites, as documented in *Geoponica (6–8).* By assessing the climatic and topographic characteristics of their lands, they were able to select the best varieties of *Vitis vinifera* L., and therefore obtain wines with particular flavors [74,92,93]. Wine consumption has been widely attested at Motya, since the earliest time of the settlement. The rich repertoire of Phoenician and Proto-Corinthian drinking vessels evidences the practice of the symposion, a social custom widely diffused in the Phoenician society [34]. In this work, biochemical analysis supported the previous archaeological evidence, revealing, in calculus samples, tartaric acid, which is one of the main metabolites of grapes and wine [94,95,96].

Fatty acids are abundant and omnipresent in animal and plant matter, although varying in quantity [48,49,50]. However, it is difficult to associate fatty acids found in calculi to specific dietary resources. Dental plaque development is a complicated biological occurrence, which has not been completely understood and depends on depositional and non-depositional factors. Dental calculus growth may affect organic residues’ absorption differently but, surely, several lipid profiles (from animal, plant, and microbiota residues) overlap each other in tartar, masking and altering the natural ratios present in their respective original sources. Additionally, exposure to cooking procedures may alter the lipid profile of foods and the content of saturated fatty acids tends to increase since the unsaturated ones are much more susceptible to degradation processes [7,47,52].

EPA and DHA, observed in some specimens, could represent traces of marine foods [97]. These polyunsaturated acids are highly sensitive to oxidative processes [98] and rarely detected in archaeological samples [99]. However, it is important to bear in mind that dental calculus is a strongly mineralized matrix which grows rapidly (about three weeks) and isolates biomolecules, protecting them from oxidative reactions. In general, fish resource exploitation (e.g., bluefin tuna and dye-murex) was certainly one of the strengths of the Phoenician economy [100,101,102]. Remains of shellfish and tuna fish have been frequently found in archaeological contexts of Motya, both in domestic and in sacred areas, where they were consumed during rituals [26,80].

The consumption of fruit, leaves, skin, and bark of a wide range of plant species, probably endemic of the Mediterranean basin (e.g., Lamiaceae and Asteraceae), and milk and dairy products was hypothesized by the detection of several plant secondary metabolites (e.g., terpenic compounds) and lactose/galactose, respectively. In Motya, consumption of milk derivatives is unsupported by archaeological records; however, what is known is that Pheonicians were makers and consumers of milk, cheese, yoghurt, and clarified butter [74]. At the studied site, bone remains of sheep and goats also suggest that these animals could be bred for meat and secondary products [80].

For centuries, plant roots and rhizomes have been considered to be valuable phytotherapeutic remedies by Ayurvedic, Greek, Arabian, Egyptian, and Roman ancient folk medicine [103,104]. However, equivalent medical evidence in the Phoenicians’ case remains undocumented. In the present research, the recovery of specific metabolites potentially linkable to plant underground tissues of *Nardostachys* sp., *Acorus* sp., and *Curcuma* sp. provides fascinating information about the pharmacognosy of the studied population, particularly given that their commercial networks extended throughout the Mediterranean. Unfortunately, a single metabolite is not enough to prove that a plant species was consumed regularly. In the Hellenistic world, the *Nardostachys* sp. was a typical ingredient of spice blends used for seasoning wine and foods, while in Indian culture it was employed as a stimulant, antispasmodic, and antiepileptic bitter drug [105]. Similarly, rhizomes and essential oils from the *Acorus* species, native to India, were used both for cooking and treating illnesses, such as asthma, dyspepsia, as well as skin and mental disorders. Chewing this plant material to alleviate toothache was also reported [106,107,108]. Finally, *Curcuma* sp., as well as being applied for medicinal purposes, including treatment of oral diseases, was used from ancient times in cosmetics, fabric coloring, and cooking [16,109]. Historical literature states that Phoenicians commonly used various types of plant species, including mushrooms (e.g., truffles), fruits (e.g., dates, figs, pomegranates, almonds, and limes), and plant underground storage organs (e.g., roots, rhizomes, and bulbs), which corroborated our archaeobotanical records [74]. In addition, interesting preliminary data obtained from carpological assemblage of Motya revealed caryopses of barley and wheat, pulses, as well as seeds and pedicels of *V. vinifera*. In addition, the anthracological remains included *Olea europaea* L. and *Quercus* sp. species, as confirmed by pollen evidence [110]. Thus, the current results seem to agree with the macro-remains found at the site; however, further analyses are necessary to fully understand this community’s paleoenvironment and paleodiet.

Our results highlight the potential of dental calculus analyses for future biomolecular studies. However, the biochemical approach on this matrix is still highly challenging, as the organic matter entrapped is variable [13]. In particular, it would be interesting to be able to specifically define both dietary and non-dietary sources for the lipid component, which remains generic.

## 4. Materials and Methods

### 4.1. Dental Calculus Collection

About 70 human skeletal remains were exhumed from the necropolis of Motya (San Pantaleo island, Sicily, Italy). Currently, these samples are hosted and preserved at Motya in superintendency storerooms. All dentitions were examined for calculus deposits; supragingival tartar was detected in only twenty-one individuals (Table 1). Mineralized flakes were removed from tooth enamel by an autoclaved dental pick and collected on an aluminum foil. The samples were placed in sterilized microcentrifuge tubes and transferred to the Department of Biology of the University of Rome ‘Tor Vergata’ (Italy) for analysis.

### 4.2. Decontamination and Sterilization Protocols

Following the suggestions of Crowther et al. [111], Gismondi et al. [112], and Soto et al. [113], a meticulous and intensive cleaning regime, using boiling sterilized water, 5% sodium hypochlorite (NaClO), 5% sodium hydroxide (NaOH), and Micronova NovaClean (Thermo Fisher Scientific), was applied for wiping surfaces, instruments, and floors of all workspaces, before conducting the laboratory contamination check. Synthetic sponges were employed to prevent plant fiber contamination. Access to the laboratories, at any time, was permitted only to specialized personnel, who wore disposable protective clothing and nonpowdered gloves. Additionally, to minimize the risk of contamination from modern starches, environmental pollutants, and airborne contaminants, fresh disposable consumables (e.g., centrifuge tubes) and instruments (e.g., glassware, microscope slides, cover slip, and metal tools) were autoclaved for 2 h, immediately prior to use. Systematically, before and after the cleaning procedures, horizontal glycerol-based slide traps were placed in the laboratories and inside the sterile vertical laminar flow hood (Heraeus HERAsafe HS12 Type), to verify the efficiency of the decontamination protocols (see results in Supplementary Material 2). Similarly, the absence of plant micro-residues in laboratory reagents and materials was also monitored.

Before proceeding with the preparation of a sample, soil still adhering to the external part of the ancient mineralized plaque was gently removed under a stereomicroscope (Leica ZOOM 2000, Leica, Buffalo, NY, USA), at a magnification of 30X, by a fine sterile acupuncture needle. Once the surface was adequately cleaned, dental calculus was subjected to the following decontamination and sterilization protocols, under a sterile vertical laminar flow hood. To remove any past and modern environmental contaminant from the calculus surface, each sample was treated with UV light for 10 min and immersed in 1 mL of 2% NaOH for 15 min. Flakes were, then, washed twice with sterile bidistilled water (40 °C), rinsed in 50 μL of 100% ethanol, and left to evaporate at 37 °C. Before and after the previous cleaning procedure, six randomly selected dental calculi were washed with sterile bidistilled water examined by optical microscopy, to confirm the efficacy of the method. No micro-debris was revealed after decontamination (Supplementary Material 3). The weight of the dental calculus collected from each individual is reported in Table 1. Each calculus sample was equally divided for performing microscopy and chromatographic analyses.

### 4.3. OM Analysis.

As reported in D’Agostino et al. [9] and Gismondi et al. [16], a decalcification treatment, using 0.5 mL of 0.2 M hydrochloric acid (HCl), was carried out for 8 h, to extract microfossils entrapped in the calculus matrix. After centrifugation for 10 min at 11,000× *g*, the pellet was washed three times with ultrapure water and mounted on a glass slide, adding a drop of mounting medium (glycerol/bidistilled water, 1:1, *v*/*v*), under a sterile vertical laminar flow hood. The samples were examined by an optical microscope (ZEISS Axio Observer 7, Zeiss, Jena, Germany) equipped with polarized filters. The size of all detected micro-debris was measured using Zen imaging software 2.6. The recovered starches’ taxonomic identity was recognized by comparing the morphology to our modern reference collection [36] and literature data and described using the International Code for Starch Nomenclature [35]. Additional scientific works and databases were consulted for the identification of the other micro-remains [37,38,39,44].

### 4.4. GC-MS Analysis

The chromatographic analysis was performed using a GC-MS QP2010 system (Shimadzu, Kyoto, Japan), equipped with a DB-5 capillary column (Phenomenex, length 30 m × diameter 0.25 mm × thickness 0.25 μm), in triplicate on each dental calculus sample, according to D’Agostino et al. [9] and Gismondi et al. [16]. Once solubilized in 0.5 mL of 3% HCl, the samples were incubated with 0.5 mL di hexane, in agitation, for two hours. After centrifugation at 11,000× *g* for 5 min, the supernatant fraction was recovered and dried out. The pellet was resuspended in 60 µL of hexane and derivatized with 40 µL of Methyl-8 Reagent (Thermo Scientific, Bellefonte, PA, USA), following manufacturers’ guidelines. Two microliters of extract were injected into the instrument at a temperature of 280 °C, in splitless modality. The carrier gas was helium, employed at a constant flow of 1 mL/min. Column temperature was initially set at 60 °C for 5 min (initial oven temperature) and, then, increased at a rate 6 °C/min to 150 °C for 5 min, to 250 °C for 5 min, and to the final temperature of 330 °C for 25 min, to obtain better resolution. An electron impact of 70 eV (scanning from 100 to 700 *m/z*) was used for the ionization (ion source temperature 230 °C, interface temperature 320 °C, solvent cut time 6 min). The peaks, or rather the detected molecules, were identified by comparing their mass spectra with those registered in the software database NIST (National Institute of Standards and Technology) Library 14 and on-line support [114]. Literature data and scientific food databases [115,116] were consulted for deducing plant species and food categories.

## 5. Conclusions

Exploring the potential of the archaeobotany on ancient human dental calculus, in the present paper, we detailed the remarkable customs of the Motya’s Phoenician community, concerning the numerous plant species which they used for subsistence. As our data refer to a limited series of individuals, it is still impossible to definitively attribute the inclusion pathways of some micro-remains in the ancient mineralized plaque, for example, breathing, cooking, and performing phytotherapy, religious, and working practices. However, microparticles hidden in tartar reveal the inextricable relationship that existed between an individual and the surrounding environment. Our research identified some aromatic and officinal herbs typical of Egypt and the Levant’s, confirming the wide reach of the Phoenicians’ commercial network. The data coming from the ancient dental calculus are consistently integrated with those available and documented by written sources and archaeological remains, providing significant insight into the lifestyle of Motya inhabitants during the eight to sixth century BC. Although the data on remains documented in this study are limited, they potentially evidence the ethnobotanical choices made by the community and highlight the strong interaction between Phoenicians and plants, in terms of cultural habits and land use.

Further investigations into plant macro-remains and soil pollen profiles at the same archaeological site could confirm and enrich the ancient framework outlined.

## Figures and Tables

**Table 1:** Micro-remains detected in dental calculus by optical microscopy (OM). Lab code, burial (identification number), sex (M, male; F, female; ND, not determined; IND, indeterminate), age at death (in years) of each individual were specified, together with the weight of dental calculus. The amount of the micro-debris (i.e., starches, pollen grains, and other particles) identified in dental calculus was reported. T, plant trichome; A, fragment of feather barbule of Anseriformes; F, unidentified fiber.

Lab Code	Burial	Sex	Age at Death	Weight of Calculus (g)	Starches	Pollen Grains	Other Micro-remains
**1**	**3**	ND	<55	0.040	1		
**2**	**4**	ND	20–25	0.020			
**3**	**8**	F	30–40	0.020		1	1A
**4**	**9**	ND	<45	0.020	9		
**5**	**10**	ND	<45	0.020			
**6**	**11**	F	20–25	0.090	3		
**7**	**12**	M	30–35	0.040		1	
**8**	**16**	ND	20–25	0.050			
**9**	**18**	ND	<30	0.060	50		
**10**	**20**	ND	35–40	0.060		1	1T
**11**	**21**	ND	30–35	0.050			1T, 2F
**12**	**23**	M	15–20	0.080			
**13**	**24**	M	35–40	0.020	2		
**14**	**25**	M	15–20	0.010			
**15**	**30**	M	30–40	0.080			
**16**	**30 02**	IND	4 ± 24	0.020			
**17**	**31**	ND	20–25	0.020			
**18**	**32**	ND	<35	0.030	1		
**19**	**33**	M	30–40	0.016			
**20**	**36**	F	25–30	0.030	3		
**21**	**38**	M	40–50	0.019		2	
	**Total**				**69**	**5**	**3**

## Supplementary Materials

The following are available online at https://www.mdpi.com/2223-7747/9/10/1395/s1. Supplementary Material 1: Molecules detected in Phoenician dental calculus by GC-MS, excluding *n*-alkanes and *n*-alkenes, Supplementary Material 2: Results of lab contamination tests by horizontal slide trap (others: fibers, hairs, dust residues), Supplementary Material 3: Optical microscopy results of the washing water applied on ancient dental calculus before the cleaning procedure.

## Abbreviations

OM	Optical microscopy
GC-MS	Gas chromatography mass spectrometry
NaClO	Sodium hypochlorite
NaOH	Sodium hydroxide
HCl	Hydrochloric acid
EPA	Eicosapentaenoic acid
DHA	Docosahexaenoic acid
