# Supplementary material for: Investigating Plant Micro-Remains Embedded in Dental Calculus of the Phoenician Inhabitants of Motya (Sicily, Italy)
Source: Plants (Basel). 2020 Oct 20;9(10):1395. doi: 10.3390/plants9101395 (PMC7590007; doi:10.3390/plants9101395)
Supplement: Supplementary file 1 [file plants-09-01395-s001.pdf]

SUPPLEMENTARY MATERIALS

Suppl. Mat. 1

Molecules detected in Phoenician dental calculus by GC-MS, excluding *n*-alkanes and *n*-alkenes.

**Burial 3**			
Sugars			Lactose
Amino acids			Asparagine
Fatty acids	Saturated		Dodecanoic acid; Tridecanoic acid; Hexadecanoic acid; Heneicosanoic acid; Triacontanoic acid
	Unsaturated	omega-3	Eicosapentaenoic acid
		omega-6	9,12-Octadecadienoic acid
Alcohols			Decanol; Tetradecenol; Tricosanol
Terpens and terpenoids	Monoterpenes and derivatives		Levomenthol; Citronellol

**Burial 4**			
Sugars			Mannose
Amino acids			Serine
Fatty acids	Saturated		Dodecanoic acid; Hexadecanoic acid; Octadecanoic acid; Heneicosanoic acid; Pentacosanoic acid
	Unsaturated	omega-6	9,12-Octadecadienoic acid; 8,11,14-Eicosatrienoic acid
Alcohols			Decanol; Methyldodecanol; Tetradecenol
Terpens and terpenoids	Monoterpenes and derivatives		Levomenthol

**Burial 8**		
Amino acids		Serine
Fatty acids	Saturated	Dodecanoic acid; Hexadecanoic acid; Octadecanoic acid; Pentacosanoic acid
Alcohols		Dodecanol; Tetradecanol; 9-Octadecenol; Heptacosanol; Triacontanol
Terpens and terpenoids	Monoterpenes and derivatives	alpha-Pinene

**Burial 9**			
Sugars			Lactose
Fatty acids	Saturated		Hexadecanoic acid; Octadecanoic acid
	Unsaturated	omega-9	9-Octadecenoic acid
Terpens and terpenoids	Monoterpenes and derivatives		Levomenthol
	Sesquiterpenes and derivatives		Carotol

**Burial 10**		
Amino acids		Serine
Fatty acids	Saturated	Butanoic acid; Hexadecanoic acid; Octadecanoic acid

	Unsaturated	omega-9	7-Hexadecenoic acid
Alcohols			Dodecanol

Burial 11		
Amino acids		Serine
Fatty acids	Saturated	Butanoic acid; Hexadecanoic acid; Octadecanoic acid; Docosanoic acid
	Unsaturated	3-Octenoic acid
Alcohols		Dodecanol; Methyldodecanol
Terpens and terpenoids	Monoterpenes and derivatives	beta-Ocimene
Phenolic compounds and derivatives		Anisole

Burial 12		
Amino acids		Alanine; Serine
Fatty acids	Saturated	Dodecanoic acid; Hexadecanoic acid; Octadecanoic acid
Alcohols		Decanol; Methyldodecanol
Terpens and terpenoids	Monoterpenes and derivatives	alpha-Pinene
	Diterpenes and derivatives	Abietic acid
	Sesquiterpenes and derivatives	alpha-Cedrene; Junenol; Salvialenone

Burial 16			
Sugars			Glucose
Amino acids			Serine
Fatty acids	Saturated		Pentanoic acid; Hexadecanoic acid; Octadecanoic acid; Tetracosanoic acid
	Unsaturated	omega-3	Docosahexaenoic acid
		omega-6	9,12-Octadecadienoic acid
Alcohols			Dodecanol; Tetracosanol
Terpens and terpenoids	Monoterpenes and derivatives		Citronellol; alpha-Pinene; Limonenol
Other markers			Sesamin

Burial 18			
Amino acids			Lysine
Fatty acids	Saturated		Butanoic acid; Hexadecanoic acid; Triacontanoic acid
	Unsaturated	omega-6	9,12-Octadecadienoic acid
Alcohols			Nonanol; Dodecanol
Terpens and terpenoids	Monoterpenes and derivatives		delta-Carene; beta-Ocimene
	Sesquiterpenes and derivatives		Spirojatamol
Other plant markers			Tartaric acid; Thiocyanic acid

Burial 20		
Fatty acids	Saturated	Hexadecanoic acid; Octadecanoic acid
Terpens and terpenoids	Monoterpenes and derivatives	Sabinol; alpha-Pinene

	Diterpenes and derivatives	Dehydroabietic acid

**Burial 21**			
Sugars			Lactose
Amino acids			Serine
Fatty acids	Saturated		Butanoic acid; Hexadecanoic acid; Octadecanoic acid; Tetracosanoic acid
	Unsaturated	omega-3	Docosahexaenoic acid
		omega-6	5,8,11,14-Eicosatetraenoic acid
		omega-9	9-Octadecenoic acid
Alcohols			Decanol
Terpens and terpenoids	Monoterpenes and derivatives		alpha-Pinene; Citronellol
	Sesquiterpenes and derivatives		Spirojatamol

**Burial 23**			
Fatty acids	Saturated		Hexanoic acid; Dodecanoic acid; Hexadecanoic acid; Octadecanoic acid
	Unsaturated	Omega-3	Docosahexaenoic acid
		omega-9	7-Hexadecenoic acid
Alcohols			Tetradecanol
Terpens and terpenoids	Monoterpenes and derivatives		Citronellol; alpha-Pinene
	Sesquiterpenes and derivatives		Cedrol

**Burial 24**			
Sugars			Glucose
Amino acids			Serine
Fatty acids	Saturated		Butanoic acid; Tridecanoic acid; Hexadecanoic acid; Octadecanoic acid
	Unsaturated	omega-9	9-Octadecenoic acid
Terpens and terpenoids	Monoterpenes and derivatives		alpha-Pinene; Citronellol
Other plant markers			Tartaric acid

**Burial 25**			
Amino acids			Serine
Fatty acids	Saturated		Nonanoic acid; Hexadecanoic acid; Triacontanoic acid
	Unsaturated	omega-3	Eicosapentaenoic acid
		omega-9	9-Octadecenoic acid
Terpens and terpenoids	Monoterpenes and derivatives		Citronellol; alpha-Pinene

**Burial 30.02**	
Sugars	Galactose; Lactose
Amino acids	Serine

Fatty acids	Saturated		Dodecanoic acid; Hexadecanoic acid; Octadecanoic acid; Eicosanoic acid; Heneicosanoic acid; Docosanoic acid; Tetracosanoic acid; Hexacosanoic acid; Heptacosanoic acid; Octacosanoic acid; Triacontanoic acid
	Unsaturated	omega-3	Docosahexaenoic acid
		omega-6	9,12-Octadecadienoic acid
		omega-7	9-Hexadecenoic acid; 11-Hexadecenoic acid
		omega-9	7-Hexadecenoic acid; 9-Octadecenoic acid
Alcohols			Decanol
Terpens and terpenoids	Monoterpenes and derivatives		Isomenthol; delta-Carene
	Sesquiterpenes and derivatives		Curdione

Burial 30			
Sugars			Galactose
Fatty acids	Saturated		Butanoic acid; Dodecanoic acid; Hexadecanoic acid; Octadecanoic acid
	Unsaturated	omega-9	7-Hexadecenoic acid
Alcohols			1-Decanol
Terpens and terpenoids	Monoterpenes and derivatives		alpha-Pinene; Citronellol
Other plant markers			Tartaric acid

Burial 31			
Amino acids			Alanine
Fatty acids	Saturated		Hexadecanoic acid; Octadecanoic acid; Tetracosanoic acid
	Unsaturated	omega-3	Eicosapentaenoic acid
		omega-6	9,12-Octadecadienoic acid
		omega-9	9-Octadecenoic acid
Alcohols			Dodecanol
Terpens and terpenoids	Monoterpenes and derivatives		alpha-Pinene
	Sesquiterpenes and derivatives		alpha-Acoradiene

Burial 32			
Sugars			Glucose
Amino acids			Serine
Fatty acids	Saturated		Dodecanoic acid; Hexadecanoic acid; Octadecanoic acid; Triacontanoic acid
	Unsaturated	omega-3	Docosahexaenoic acid
		omega-9	9-Octadecenoic acid
Alcohols			Undecanol
Terpens and terpenoids	Monoterpenes and derivatives		delta-Carene
Phenolic compounds and derivatives			6-Methyloctahydrocoumarin

Burial 33			
Amino acids			Serine
Fatty acids	Saturated		Butanoic acid; Octadecanoic acid

	Unsaturated	omega-9	7-Hexadecenoic acid
		omega-12	6-Octadecenoic acid
Terpens and terpenoids	Monoterpenes and derivatives		Citronellol; alpha-Pinene
	Sesquiterpenes and derivatives		Sesquicineole
Other markers			Orotic acid; Tartaric acid

Supp. Mat. 2

Results of lab contamination tests by horizontal slide trap (others: fibres, hairs, dust residues).

Context	Location of traps (number)	Starches	Pollen grains	Others	Total
**Workday**	workbench (10)	1	2	0	**3**
	floor (10)	5	3	2	**10**
	shelf (5)	2	1	0	**3**
	hood (5)	0	0	0	**0**
**After decontamination**	workbench (10)	0	0	0	**0**
	floor (10)	1	0	0	**1**
	shelf (5)	1	0	0	**1**
	hood (5)	0	0	0	**0**

Suppl. Mat. 3

Optic microscopy results of the washing water applied on ancient dental calculus before the cleaning procedure.

**Burial**	**Polyhedral starches**	**Partially gelatinized starches**	**Indeterminate particle**	**Lamiaceae pollen grains**	**Rosaceae pollen grains**	**Fungal spores/hyphae**	**Plant fibres**	**Hairs**
**8**	2	1		1			1	
**11**			1			1		1
**21**							2	
**31**	1		3				2	
**32**		1			1			
**38**								
